# Fast all versus all genotype comparison using DNA/RNA sequencing data: method and workflow

**DOI:** 10.1186/s12859-023-05288-y

**Published:** 2023-04-24

**Authors:** Steven A. Eschrich, Xiaoqing Yu, Jamie K. Teer

**Affiliations:** Department of Biostatistics and Bioinformatics, H. Lee Moffitt Cancer Center and Research Institute, Tampa, FL USA

**Keywords:** Massively parallel sequencing, Quality control, Genotype comparison

## Abstract

**Background:**

Massively parallel sequencing includes many liquid handling steps which introduce the possibility of sample swaps, mixing, and duplication. The unique profile of inherited variants in human genomes allows for comparison of sample identity using sequence data. A comparison of all samples vs. each other (all vs. all) provides both identification of mismatched samples and the possibility of resolving swapped samples. However, all vs. all comparison complexity grows as the square of the number of samples, so efficiency becomes essential.

**Results:**

We have developed a tool for fast all vs. all genotype comparison using low level bitwise operations built into the Perl programming language. Importantly, we have also developed a complete workflow allowing users to start with either raw FASTQ sequence files, aligned BAM files, or genotype VCF files and automatically generate comparison metrics and summary plots. The tool is freely available at https://github.com/teerjk/TimeAttackGenComp/.

**Conclusions:**

A fast and easy to use method for genotype comparison as described here is an important tool to ensure high quality and robust results in sequencing studies.

## Background

The continuing decrease in massively parallel or next-generation sequencing (NGS) costs has enabled large projects consisting of hundreds or thousands of samples. Although sample and sequencing library preparation protocols have improved and are often automated, there are many sample manipulations and considerable human input required. This leads to the chance of sample integrity issues, including sample swapping, mixing, duplication, and sequencer lane assignment errors [1]. Human genotype comparison at common inherited variant positions can determine the degree of genetic difference between samples. Samples from the same individual should have a variation rate near zero, whereas samples from unrelated individuals will have higher variation rates. Confirmation of sample identity has become an important quality control step in NGS experiments.

Sample quality control via genotyping comparison is most informative when relationships between samples exist: tumor-normal matched pairs for somatic mutation detection, family studies to identify rare variants, and multi-omics studies with DNA and RNA sequencing from the same patient. Matching pairs can be confirmed using variant comparison to ensure they come from the same (or a related) individual. Indeed, pairwise comparison tools like Genome Analysis Toolkit (GATK) GenotypeConcordance [2] and IDcheck [3] allow for such an analysis. However, even when no relationships are known to exist, we have uncovered cases in which samples show genotype concordance even though they should not. Comparing each sample in a sequencing project to all other samples can reveal unexpected genetic similarity due to sample handling issues, hidden relatedness, patient re-enrollment, etc. In addition, sequencing experiments can include samples from the same individual where some genetic differences are expected, such as in matched tumor/normal tissue comparisons. An all vs. all comparison provides a more complete range of genotype discordances, so a matched pair with non-zero discordance is easily distinguished from the greater discordance of samples from different individuals.

Confirming sample relatedness is an important consideration for family studies, and several tools exist that leverage common pedigree file formats. These tools allow for all vs. all comparisons to ensure relatedness is as expected (HYSYS [4], NGSCheckMate [5], Peddy [6]). However, these tools may require specific input formats describing relatedness structures, and it is not always clear how well they will scale to larger cohorts. Here we assume no relatedness structure and use after-the-fact inference to determine associations/sample swaps. We have developed a Perl tool to rapidly compare genotypes from thousands of samples in an all vs. all manner. The key optimization for rapid comparison is the use of bitwise representation and operations. An end-to-end Workflow Descriptor Language (WDL)/Cromwell workflow taking FASTQ, BAM, or VCF files as input was developed for reproducibility and ease of use. The workflow, TimeAttackGenComp, is publicly available at https://github.com/teerjk/TimeAttackGenComp under the 3-clause Berkeley Software Distribution (BSD) license.

## Results

Our goal is to measure genotype discordance between samples across all pairwise sample combinations. We start by defining a region of positions to query as a BED file. Although any positions can be defined, common human variant positions will be the most informative. We used 1000 Genomes positions with population allele frequency ≥ 15% in protein coding regions for this study as they are likely to be covered in Whole Exome Sequencing and RNAseq experiments. Single nucleotide variant genotype information for each sample at each position in the region is stored in memory as either the reported genotype or a missing value. Genotypes are internally encoded as single upper-case characters as defined by the International Union of Pure and Applied Chemistry (IUPAC), and missing or low-quality genotypes are encoded as the American Standard Code for Information Interchange (ASCII) text NULL character (\0, decimal value of 0). All genotypes for a sample at all positions in the region are then stored as a concatenated string, allowing for full precision in the region of interest.

**Fig. 1:**
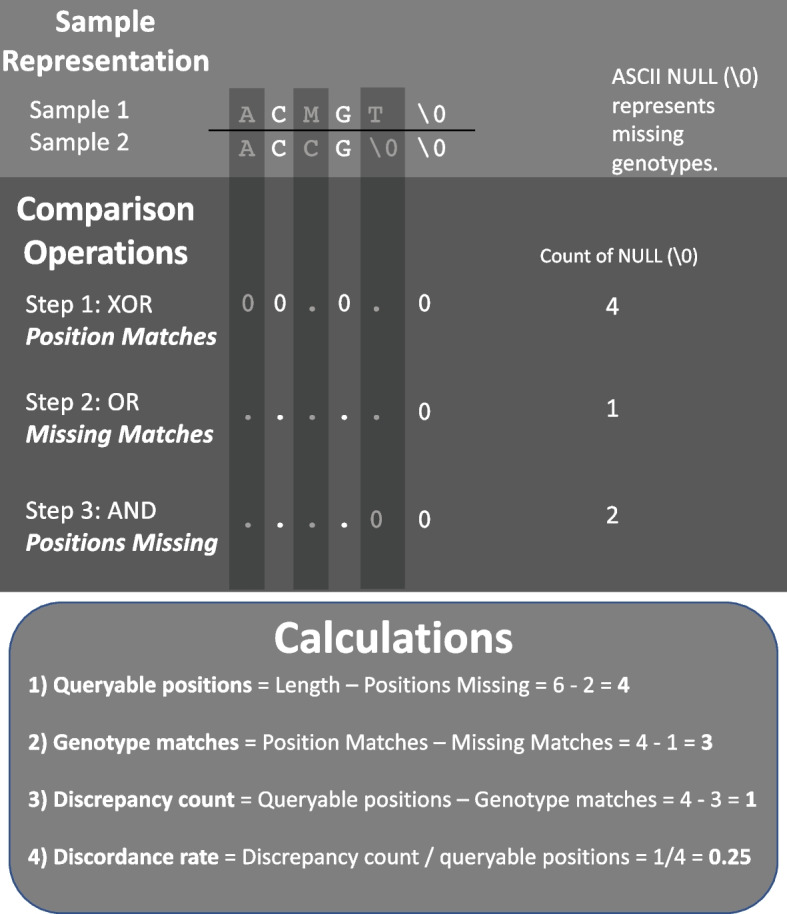
Overview of bitwise comparison algorithm. \0 indicates a value of zero, which in ASCII is the NULL character. Steps 1–3 describe the bitwise operation used between the two genotype strings, and result values of 0 (NULL character) are counted. A period (.) is used to represent a non-zero value, which is not counted

To achieve fast performance the genotype comparison itself is performed using bitwise string operations on the stored genotype strings as illustrated in Fig. 1. Perl performs bitwise operations on each byte of a string. By using single character ASCII representations of our genotypes, we can compare genotypes at all desired positions more efficiently. Three binary Boolean operations (AND, OR, XOR) are used to derive comparisons and the Perl transliteration operator is used in scalar context to count bitwise byte comparison results of NULL. In our comparisons, we only count instances of the NULL value; non-NULL results are ignored. Pairwise comparisons are performed once for each possible sample pair. In Step 1 (Position Matches), the total number of matching genotypes is counted by performing an XOR operation on the genotype strings and counting the resulting NULL. Positions at which both samples are missing (Missing Matches) are counted by performing an OR operation and counting NULL characters that only result from comparing two missing (\0) values (Step 2). We next calculate the total number of queryable positions where neither sample has a missing (NULL) value (Step 3, Positions Missing). Since genotypes are encoded as upper-case ASCII, all non-missing genotypes have bit 6 set ensuring an AND operation will return NULL only when at least one genotype is missing (Step 3). The number of queryable positions is then calculated by subtracting the Positions Missing count from the total genotype string length (Eq. 1). Genotype matches also include positions where both samples have a NULL genotype (both are missing data), so Missing Matches are subtracted from Position Matches to give the true number of Genotype matches (Eq. 2). Discrepant positions are counted by subtracting the genotype matching count from the total number of queryable positions (Eq. 3). The discordance rate is finally calculated by dividing the discrepancy count by total queryable positions (Eq. 4).

1 $${\textit{Queryable\,positions}} = \left( {{\textit{Length}} - {\textit{Positions\,Missing}}} \right)$$

2 $${\textit{Genotype\,matches}} = \left( {{\textit{Position\,Matches}} - {\textit{Missing\,Matches}}} \right)$$

3 $${\textit{Discrepancy\,count}} = \left( {{\textit{Queryable\,positions}} - {\textit{Genotype\,matches}}} \right)$$

4 $${\textit{Discordance\,rate}} = \frac{{\textit{Discrepancy\,count}}}{{{\textit{Queryable\,positions}}}}$$

**Fig. 2:**
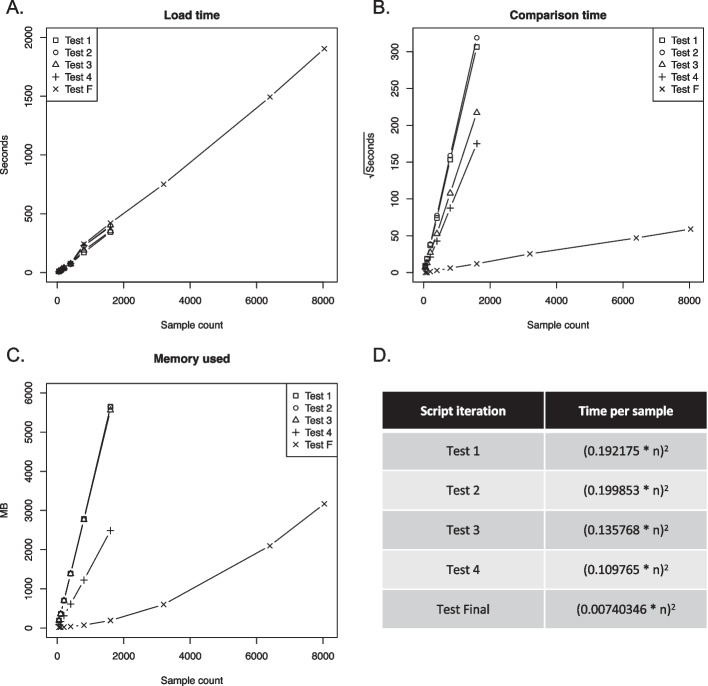
Performance of different approaches to genotype comparison. Test description: (1) two-way string comparison using array copying (pass-by-value), (2) array referencing (pass-by-reference), (3) one-way string comparison (sample A vs. sample B), (4) one-way numeric index comparison with genotypes encoded as integers, (F) our one-way bitwise approach. **A** Time to load versus sample count. **B** Time to perform all comparisons versus sample count. Note the y-axis is plotted as a square-root transform. **C** RAM used during comparison versus sample count. **D** Linear models of time per sample of the different approaches

Our initial approach to genotype comparison was a simple two-way string comparison (performed in both directions; sample A vs. B and sample B vs. A). However, this approach did not scale well with increasing sample sizes. We compared two-way string comparisons using array copying (pass-by-value) (Test 1), array referencing (pass-by-reference) (Test 2), one-way string comparison (sample A vs. sample B) (Test 3), one-way numeric index comparison (genotypes encoded as integers, Test 4), and finally our one-way bitwise approach (Test F). Testing was performed on a high performance compute cluster node (2 × Intel Xeon E5-2470 2.3 GHz, 165 GB RAM, perl 5.10). Results were compared after each test to ensure they were the same. Sample loading time, comparison time, and memory use were measured with increasing sample numbers. Sample loading time is proportional to the total number of samples (N), with some methods showing a slight increase above a certain N (Fig. 2a). While the original string comparison (Test 1) showed reasonable comparison time with sample counts up to around 100, comparison time increases as a function of N^2^ (Fig. 2b). Unsurprisingly, a one-way comparison (Test 3) reduced time to almost half. Numeric index comparison reduced time an additional amount compared to string comparison. However, bitwise comparison of genotype strings reduced comparison time dramatically, and allowed an all vs. all comparison of 1600 samples in 143 s (Test F) compared to 26 h for the two-way string comparison (Test 1). When it became impractical to apply our earlier approaches to a large dataset of 8037 samples, the bitwise method (Test F) took 58.2 min. Memory usage was also decreased in the bitwise approach: comparison with 8037 samples used just over half the memory of the string-comparison methods with only 1600 samples (Fig. 2c). Performance time (Fig. 2d) was modelled based on linear regression of square root time in Fig. 1b. Finally, we ran the final bitwise comparison algorithm (Test F) on 8037 samples using a 2019 MacBook Pro (Intel Quad-Core i5 2.4 GHz, 16 GB RAM, perl 5.30). Performance was slightly better than that observed on the HPC: load time = 20.5 min, comparison time = 45.3 min, memory usage = 2.6 GB.

## Implementation

Quality control is an essential part of DNA/RNA sequencing analyses, but tool-specific input format requirements may result in users ignoring this step. We have therefore packaged our tool in an end-to-end workflow designed to start with either raw FASTQ, aligned BAM files, or genotype VCF files and produce a summary output matrix and heatmap of pairwise genotype discordance values. This workflow is written in WDL (https://openwdl.org) and was tested with the Cromwell execution engine (https://github.com/broadinstitute/cromwell). Sequence alignment of raw FASTQ files is performed using the SNAP rapid aligner (https://arxiv.org/abs/1111.5572), and genotype calling utilizes samtools bcftools [7]. A VCF-converter prepares the genotypes for comparison, and the comparison is performed with the Perl tool described above. Tasks are also included to extract and plot allele frequency information for the genotype calls. Plotting is performed in R (https://www.r-project.org/).

**Fig. 3:**
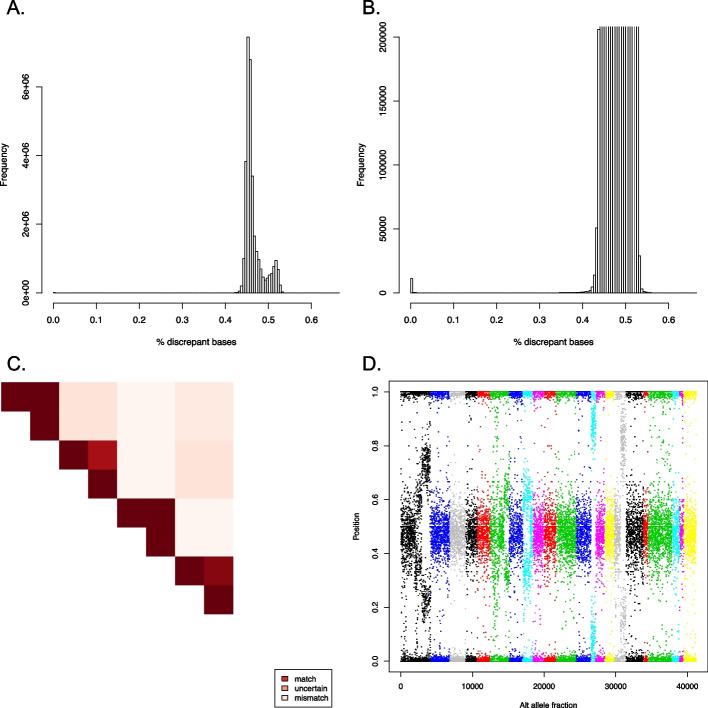
Comparison output examples. **A** Distribution of discordance rates between samples. **B** Zoomed y-axis of sample discordance distribution, illustrating low discordance of samples from the same individuals. The number of low discordance comparisons is low as most comparisons are between samples from different individuals in this example. **C** Example heatmap highlighting lower discordance (dark red) between samples from the same individuals. In this example, every two samples belong to the same individual, and the immediate off-axis dark-red indicates low discordance between these matching samples. **D** Example allele frequency plot of a single sample, colored by chromosome

We have used this workflow extensively to perform quality control on a variety of sequencing projects. This includes Whole Exome Sequencing (WES) projects with multiple samples for each individual, as well as projects with WES and RNAseq from each individual. We have detected instances where samples that are reported as being from the same person do not match genetically, and also instances where samples reported as not being from the same person do match genetically. The all vs. all analysis provides the ability to identify where similarities and differences exist. In some cases it was clear that a sample swap occurred, and further investigation justified correction of the sample swap. In other cases, no apparent swap occurred, but problematic samples were identified for resequencing. Figure 3a and b (zoomed view) illustrates the distribution of discordance values across 8037 samples. Discordances between samples from different individuals range from 40 to 55%, while samples from the same individual (the minority of comparisons in this example) range from 0 to 3%. However, we note that discordance rates between samples from the same individual can vary across experiments. The workflow also plots a heatmap of discordances, allowing easy visualization of inappropriate matching when samples are grouped by individual in the input file (Fig. 3c). Finally, allele frequency plots at the region of interest positions are plotted (Fig. 3d) which may help identify sample contamination, copy number variations, or other chromosomal aberrations. Although the actual genotype comparison can be run on a modern desktop or laptop computer, sequence alignment and genotype calling have higher memory and compute requirements, and we recommend running the complete pipeline on an HPC. The main limitation in the number of samples able to be compared on either HPC or local computers is based on the available free RAM (Fig. 2c).

## Conclusions

Genotype comparison across samples at inherited polymorphic positions has become an important part of NGS quality control. This has been used to compare samples that should come from the same individual. We find it useful as a general tool for any experiment to confirm samples were not duplicated or otherwise mis-handled. All vs. all comparison is very powerful as this approach allows identification of samples that should match (but don’t) and samples that should not match (but do). We also find this approach helpful in resolving sample swaps by allowing identification of the unknown samples that match the mismatching pair. However, all vs. all is an O(n^2^) problem, and scalability is an issue for larger projects. We increased the performance of genotype comparisons using low-level bitwise operations to speed up the bottle neck operation of genotype comparison. By leveraging Perl’s string bitwise operations, we were able to achieve dramatic speedup of ~ 650× as compared to string comparisons. Further improvements are likely possible. Future potential optimizations could include multi-threaded parallelization, less exact approaches, and more sophisticated approaches leveraged for sequence alignment, including clustering [8] and minimizer [9] techniques. Memory usage could be further improved with bit-packing, bit-vectors, and the use of lower-level languages. Interestingly, although much effort can be devoted to decreasing algorithmic complexity, we have found value here in optimization within a scripting language to greatly reduce the constant in this n^2^ approach. Of course, despite these efficiencies, novel algorithms will eventually be needed to reduce the complexity of this problem as scales continue to increase. Even given this eventuality, we find that leveraging low level operations available in scripting languages offers dramatic performance improvements allowing for thorough sample comparisons in large projects.

## Availability and requirements

Project name: TimeAttackGenComp.

Project home page: https://github.com/teerjk/TimeAttackGenComp

Operating system(s): Linux (may run on other platforms via containerization).

Programming language: Perl, WDL.

Other requirements: A WDL execution engine (i.e., Cromwell) and container application (i.e., Docker) are required to run the workflow. A Perl interpreter is required to run the genotype comparison tool.

License: 3-clause BSD.

Any restrictions to use by non-academics: none beyond the BSD license requirements.

## Data Availability

The software described is available without cost here: https://github.com/teerjk/TimeAttackGenComp/

## Abbreviations

NGS: Next-generation sequencing (massively parallel sequencing)
DNA: Deoxyribonucleic acid
RNA: Ribonucleic acid
GATK: Genome analysis toolkit
WDL: Workflow descriptor language
BSD: Berkeley software distribution
IUPAC: International union of pure and applied chemistry
ASCII: American standard code for information interchange
WES: Whole exome sequencing
N: Number of samples
